# Joint Developmental Trajectories of Conduct Problems and Hyperactivity/Inattention: Antecedent Risk Markers for Group Membership

**DOI:** 10.1007/s10578-023-01614-w

**Published:** 2023-11-01

**Authors:** Hannah Mercedes Araminta Ross, Lisa-Christine Girard

**Affiliations:** 1 Lancaster University, Lancaster, UK; 2 University of Oslo, Oslo, Norway

**Keywords:** Externalising problems, Conduct problems, Hyperactivity/inattention, Joint trajectories, Cohort study, Childhood

## Abstract

This study investigated joint trajectories of conduct problems and hyperactivity/inattention from age three to nine in a cohort of 7,507 children in Ireland (50.3% males; 84.9% Irish). The parent-reported Strengths and Difficulties Questionnaire was used to collect information on conduct problems (CP) and hyperactivity/inattention (HI). Information regarding risk markers was collected when participants were nine-months-old via parent report and standardised assessments. Using a person-centred approach (i.e., group-based multi trajectory modelling), six trajectories were identified: no CP/low HI, low-stable CP/HI, low-declining CP/stable HI, desisting co-occurring CP/HI, pure-increasing HI, and high chronic co-occurring CP/HI. Specific risk markers for group membership included: male sex; birth complications; perceived difficult temperament; lower primary caregiver age and education level, and higher stress level; prenatal exposure to smoking, and indicators of lower socioeconomic status. Primary caregiver-child bonding and having siblings were protective markers against membership in elevated groups. Results suggest support for both ‘pure’ HI and co-occurring trajectories of CP and HI emerging in toddlerhood. However, no support was found for a ‘pure’ CP trajectory, which may support the suggestion that children on a persistent CP trajectory will have coexisting HI. Intervention efforts may benefit from starting early in life and targeting multiple risk markers in families with fewer resources.

## Introduction

Conduct problems (CP) and hyperactivity/inattention (HI), often referred to as externalising behaviours, are estimated to affect between 7–10% and up to 20% of children, respectively [1–3]. Conduct problems and HI have been found to be the first and third most common presentations to child and adolescent mental health services in Ireland [4]. Conduct problems and HI that emerge in toddlerhood and persist through childhood and adolescence have been associated with pervasive adverse outcomes across the lifespan. For example, CP and HI have been associated with lower social competence and peer exclusion, lower educational attainment, poor mental health outcomes, criminality, and reduced political engagement, amongst others [5–12]. Furthermore, there is a high degree of co-occurrence between CP and HI in childhood, which is often associated with more severe and persistent problems and related adverse outcomes [13].

Given the high personal and social costs, along with the rising prevalence of CP and HI [14], there is an urgent need for effective early intervention efforts to support children and families with these difficulties. In order to design effective intervention programmes, it is first necessary to better understand the origins and progression of CP and HI and their potential co-occurrence, along with any risk or protective factors which may contribute to their development and/or persistence/desistence in childhood [15]. Moreover, understanding potential heterogeneity between “pure” and co-occurring trajectories of CP and HI across toddlerhood and childhood could help to better inform such efforts. Longitudinal research modelling developmental trajectories of CP and HI separately in childhood has significantly helped to develop our understanding of their aetiology and developmental course. However, with notable exceptions, there are limited studies that have specifically examined joint developmental trajectories of CP and HI from toddlerhood through to childhood, using a person-centred approach, whilst examining associated risk/protective markers for trajectory group membership [16–20]. Given the high co-occurrence found between CP and HI (e.g., [21]), this is an important endeavour in furthering our understanding. Therefore, this study aimed to model joint developmental trajectories of CP and HI, and to identify child, parent, and family characteristics which are associated with membership in trajectory groups with the most elevated levels of CP and HI.

## The Development of Conduct Problems

Conduct problems are characterised by behaviours that breach social conventions and others’ rights, including physical and indirect aggression, and non-violent disruptive behaviours, such as defiance and delinquency [22]. Moffit’s developmental taxonomy of antisocial behaviour [23, 24] proposes that a small number of children who exhibit antisocial behaviour (behaviours which characterise CP) will follow a life-course persistent trajectory, with persistent CP emerging in early childhood and escalating across the life-course. These problems are argued to develop and be entrenched through interactions between early neuropsychological vulnerabilities, such as difficult temperament and hyperactivity, and environmental risk factors, such as socioeconomic adversity and disrupted family relationships; pointing to the importance of taking a multifactorial approach when examining risk factors for CP. A child-limited trajectory of antisocial behaviour has also been suggested by the taxonomy. This group is argued to have exposure to similar early risk factors as the life-course persistent group and develop CP in early childhood; however, these behaviours should decrease to low or moderate levels in adolescence. Finally, a larger group of children, without exposure to early risk factors, are suggested to follow an adolescent onset trajectory. Their delinquent behaviour is theorised to emerge during puberty as an attempt to demonstrate maturity and independence to caregivers and peers. These behaviours are then thought to dissipate in young adulthood. Membership in the life-course persistent and child-limited groups have been linked to an increased risk of multiple adverse outcomes, including school failure, psychological and interpersonal difficulties, and criminal convictions, compared to the adolescent onset group, with those with life-course persistent difficulties at greatest risk [25].

Numerous studies using person-centred modelling approaches to identify latent developmental trajectories of CP in childhood have in part supported this taxonomy, finding small groups with persistently high levels of CP analogous with the life-course persistent group and larger group(s) with declining difficulties similar to the proposed child-limited group [26–33]. Some studies including older children and adolescents also find groups whose engagement in CP-related behaviours increases, although this generally begins prior to adolescence, somewhat different to the theorised adolescent-onset group [30, 34–36]. Consistent with Moffitt’s taxonomy, studies have shown that trajectory groups with elevated levels of CP, particularly groups with high levels of CP that remain consistently elevated over time, are at greatest risk of adverse outcomes in adolescence and adulthood [37].

The common finding of larger groups exhibiting decreasing trajectories of CP across childhood suggests that moderate but decreasing engagement in CP in early life may be a normative part of development, similar to Tremblay’s proposed early childhood perspective of aggression (e.g., [38]). For example, it is argued that physical aggression emerges alongside advances in motor control, starting in infancy and toddlerhood, but becomes “unlearnt” as further advances in brain maturation, expressive language, and social learning (which support children to inhibit these behaviours, find alternative solutions to problems, and internalise behavioural expectations) are made [32, 39]. Increasing trajectories, when found, appear to be associated with behaviours such as alcohol consumption and truancy [36, 40], which appear consistent with the suggestion that behaviours that rise in adolescence may be an attempt to display independence and impress peers [23].

## The Development of Hyperactivity and Inattention

Hyperactivity-impulsivity relates to difficulties inhibiting behaviour, with behavioural manifestations including fidgeting, interrupting, talkativeness, and risk-taking [41]. Inattention involves difficulties regulating and sustaining attention, resulting in behaviours including disorganisation, forgetfulness, and distractibility [41]. Previous studies examining trajectories of hyperactivity-impulsivity have found low (25–91%), moderate-declining (16–54%), and high trajectory groups (9–26%), with peak levels occurring between three to five years [2, 42–46]. Additional trajectories found in some studies include high-remitting (19–32%) and moderate-stable groups (25–33%) [45, 46]. Similarly, studies of inattention consistently find groups with low (9–86%), high-stable (18–23%), and moderate difficulties (33–60%), although peak levels occur slightly later, between six to eight years [2, 45, 47]. Other trajectories of inattention found include high-decreasing (18.4%), high-increasing (14%), moderate-decreasing (23%), and low-increasing (15.3%) [44–47]. Not unlike CP, membership in elevated trajectories increases the risk of adverse outcomes, including significantly lower school adjustment and prosocial behaviour in childhood, higher rates of CP, arrests, school dropout, mental health difficulties, and unemployment in adolescence and adulthood [44, 48, 49].

These findings suggest that mild to moderate HI may be normative in early childhood but typically declines around entry to school. It has also been suggested that declines may occur as children learn to inhibit hyperactive responses and engage in self-directed behaviour due to brain maturation, language development, and socialisation in early life, which facilitate the development of executive functioning. Barkley’s model of Attention Deficit and Hyperactivity Disorder [50–52], posits that persistent hyperactivity arises from neurobiological deficits that disrupt the development of behavioural inhibition. As behavioural inhibition is thought to provide a pause between stimulus and response, this leads to secondary deficits in other executive functions as there is no space to use them. Additionally, as behavioural inhibition is involved in the ability to resist distraction, these difficulties may also manifest as inattentive behaviours [51]. The later peak of inattention may be driven by certain behaviours which are thought to require more complex executive functioning, including making thoughtless mistakes, forgetfulness, losing things, and distractibility, which appear to increase between the ages of three to six [53].

## The Development of Co-occurring CP and HI

Although often studied as distinct constructs, CP and HI commonly co-occur [21]. For example, a meta-analysis including over 24,000 participants found that 24% of boys and 33% of girls with externalising problems presented with co-occurring CP/HI [13]. Furthermore, these children were found to have had the most severe and persistent behavioural difficulties and the highest risk of adverse outcomes compared to children without co-occurring CP/HI difficulties, including being more likely to engage in violent behaviour and having mental health problems as adolescents [13, 54]. From a theoretical perspective, high levels of co-occurrence could be expected, as early hyperactivity is proposed as a risk factor for life-course persistent CP [23]. However, studies that model CP and HI separately often do not account for this increased likelihood of co-occurrence, potentially obscuring developmental patterns and the specific risk factors for pure versus co-occurring problems. This further demonstrates the importance of examining the joint development of CP and HI longitudinally.

Studies which have modelled individual developmental trajectories of CP and HI, and then examined joint conditional probabilities of group membership to investigate co-occurrence between CP and HI, have found that 81–91% of children following high HI trajectories did not follow the same CP trajectory [11, 19, 55]. However, all children with high CP in the majority of these studies had at least moderate levels of HI, with 55% of boys and 96% of girls with high CP also following a high HI trajectory [19, 55]. These findings suggest that HI is likely a risk factor for CP, supporting Moffitt’s [23] taxonomy. More recently, joint trajectories of HI, non-compliance, and physical aggression were modelled in a nationally representative Canadian cohort followed from one to five years, using a person-centred approach (*n* = 2,045) [42]. Fourteen distinct groups were found. Most children (72%) followed joint trajectories characterised by a combination of low or moderate levels of each behaviour. Groups with a mix of high-persistent and moderate trajectories comprised 21% of the sample, whilst a small group of children followed high trajectories across all behaviours (7%). This would suggest that elevated difficulties in one area may translate into elevated difficulties in other areas when considering early developmental periods. Given the relative dearth of studies examining joint developmental trajectories of CP and HI starting in the toddler years through to childhood, there is a need for further studies jointly modelling these behaviours longitudinally to further develop our understanding of co-occurring CP and HI.

## Antecedent Risk Markers

Numerous child, parent, and family-related factors have been implicated in the development of CP and HI. For example, boys have consistently been found to have a greater risk of CP and HI [2, 27, 28, 47, 56], as have children with “difficult” temperaments characterised by negative affect, high reactivity, difficulty soothing, and low adaptability to change [35, 57]. Perinatal factors such as prenatal exposure to nicotine, birth complications, low birth weight, and prematurity have also been identified as risk markers for CP and HI [28, 34, 35, 58]. At the family level, maternal depression, lower maternal age, maternal education level, and maternal minoritised ethnicity have been found to be associated with group membership to trajectories characterised by high levels of CP [27, 28, 31, 35] and HI [2, 47]. Wider family characteristics, including lower socioeconomic status (SES) and larger family size, also confer risk for elevated trajectories of CP [32] and HI [44], although several studies have found that SES was not associated with HI in their sample [2, 47, 48].

Several other psychosocial factors which may confer risk remain relatively underexplored. For example, whilst maternal depression has been associated with CP and HI (e.g., [16, 58]), there is a more limited evidence base examining paternal depression. Meta-analytic findings suggest a small but significant positive association (*r* = 0.15) between paternal depression and externalising behaviour in children [59]. However, few developmental trajectory studies have examined it as a risk marker. Additionally, lower levels of maternal-child bonding, the affective bond or tie the mother feels towards their child, have been associated with elevated childhood CP trajectories [34]. However, the role of maternal bonding in HI and paternal bonding in both HI and CP is under-researched. Further, the impact of parental stress as a risk marker for CP and HI remains unclear, despite recent support linking maternal stress levels to childhood trajectories of CP and HI (e.g., [58]). Therefore, this study contributes to the existing evidence base regarding risk markers for CP and HI by attempting to replicate associations with those commonly found in past research and further extend it by considering additional potential parental psychosocial risk markers associated with joint trajectories of CP and HI.

## Aims and Hypotheses

Building on the existing evidence base, the current study aims to examine the heterogeneity in joint developmental trajectories of CP and HI from toddlerhood to childhood, whilst examining child, parental, and family-level risk markers associated with membership in elevated trajectory groups. Drawing on the literature, it is hypothesised that a five-trajectory group model will best fit the data. More specifically, it is expected that the following groups will be identified: a low CP/HI group; a normative group with early moderate CP and HI at three years which decreases to low levels of CP and HI between five and seven years of age respectively; a group with moderate-stable pure HI; a group with moderate-stable or increasing pure CP; and finally a group with co-occurring, chronic elevated levels of CP and HI (e.g., [11, 19]). It is anticipated that the low and normative groups will comprise the majority of the sample. It is further anticipated that children will be at highest risk for membership in elevated trajectories if they are boys who have been exposed to prenatal smoking; are perceived to have a difficult temperament by their caregiver; experienced birth complications, including premature delivery, low birth weight or a stay in the neonatal intensive care unit; and who were born to younger mothers, who have received less education, and have lower SES. Additionally, it is hypothesised that having one or more parents with higher postnatal stress, depressive symptoms, and lower parent-child bonding will increase the risk of membership in elevated groups.

## Methods

### Participants and Procedure

This study uses data from the Growing Up in Ireland (GUI) Infant Cohort, a national longitudinal study examining the influence of biopsychosocial factors on child development in the Republic of Ireland [60]. The Child Benefit Register was used to select a random sample of children. Child benefit is a universal benefit payable monthly to all families with children under 16 and, therefore, the register was used to ensure a representative sample of all eligible families. Families of 16,136 infants were invited to participate, of which 11,134 agreed and first participated between September 2008-April 2009, when infants were nine months old (T1, response rate: 65%). Follow-up data was collected in toddlerhood and childhood when the infants were aged three (T2, *n* = 9,793), five (T3, *n* = 9,001), seven/eight (T4, *n* = 5,344), and nine years old (T5, *n* = 8,032). At T1–T3 and T5 data was collected from primary (PCG, 99.6% mothers) and secondary caregivers, where resident (SCG, 90.6% fathers), through a home-based computer-assisted interview with trained researchers and an additional self-report sensitive questionnaire. A shorter self-report postal survey was sent to PCGs only at T4. Attrition and item non-response are common across longitudinal cohort studies. In the current study this resulted in children of younger PCGs, with lower education levels, from families with lower SES being under-represented by T5. Therefore, calculated weights were used with a sample of 7,507 participants for whom data was available across T1–T3 and T5, to ensure that participant characteristics were as close to the original nationally representative sample as possible (i.e., within 0.5% of the original participant characteristics distribution). For further description of the calculated weights, please see Quail et al. [61]. Descriptive statistics of the included sample (*n* = 7,507) are reported in Table 1. Ethical approval for the GUI cohort was obtained from the Department of Health and Children in Ireland. Written informed consent was collected prior to each round of data collection. This study was also granted ethical approval from the University of Edinburgh Ethics Committee and adhered to the British Psychological Society’s Ethical Research Guidelines [62].

**Table 1:** Sample demographic characteristics

	*N* = 7,507 (%)
Child sex	
Male	3779 (50.3)
Female	3728 (49.7)
Premature	
No	7038 (94.0)
Yes	449 (6.0)
Low birth weight	
No	7021 (94.6)
Yes	399 (5.4)
Neonatal care	
No	6472 (86.3)
Yes	1,030 (13.7)
PCG age	
16–24	627 (8.4)
25+	6880 (91.7)
PCG education	
No education/primary school	119 (1.6)
High school	2882 (38.4)
Tertiary education	4502 (60.0)
PCG ethnicity	
Irish	6354 (84.9)
Other white	772 (10.3)
Black	170 (2.3)
Asian	163 (2.2)
PCG depression	
No	6709 (90.5)
Yes	708 (9.6)
SCG depression	
No	5864 (96.3)
Yes	226 (3.7)
Siblings	
No	2915 (38.8)
Yes	4592 (61.2)
Prenatal smoking exposure	
No	5129 (69.6)
Yes	2237 (30.4)
Medical card status	
Full	1597 (21.3)
GP only	212 (2.8)
Not covered	5695 (75.9)
Social class	
Professional/managerial	4063 (54.1)
Non-manual/skilled manual	2222 (29.6)
Semi-skilled/non-skilled manual	609 (19.8)
Never worked	613 (8.2)

	M (SD)
Difficult temperament	14.71 (4.86)
PCG stress	31.83 (6.72)
PCG quality of attachment	42.55 (2.58)
SCG stress	30.84 (6.27)
SCG quality of attachment	24.09 (1.45)

*Note:* Premature status was defined as delivered prior to 37 weeks, low birth weight as < 2,500 g, and prenatal smoking exposure as whether one or more household members smoked during the pregnancy. Caregiver depression was measured using the Centre for Epidemiological Studies Depression Scale (8-item; [63]), dichotomised with a cut-off of ≥ 7. Social class was dichotomised into professional/managerial and non-professional managerial which included non-manual/skilled manual, semi-skilled/non-skilled manual and never worked. Difficult child temperament was measured by the fussy-difficult subscale of the Infant Characteristics Questionnaire [64]; caregiver stress was measured using the Parenting Stress Scale [65]; caregiver quality of attachment was measured using the Quality of Attachment subscale of the Maternal Postnatal Attachment Scale [66]

### Measures

CP and HI were measured using the five-item CP and five-item HI subscales of the Strengths and Difficulties Questionnaire, parent version (SDQ; [67]) completed by PCGs at T2-5. Respondents rate each item on a three-point scale (0 = *not true*, 1 = *somewhat true*, 2 = *certainly true*) with total subscale scores ranging from 0 to 10. The CP subscale includes items such as *“Often fights with other children or bullies them”.* The HI subscale includes items such as *“Constantly fidgeting or squirming”*. Internal consistency in the GUI sample ranged from adequate to good (i.e., CP 0.56–0.59 and HI 0.75–0.80) [60, 68]. Using previously suggested cut-offs, scores on each subscale were qualitatively categorised when describing the trajectories as follows: CP as low (0–2), moderate (3), and high (4–10), and HI as low (0–4), moderate (5–6), and high (7–10) [69], although the entire range of scores were used in the trajectory estimation. Spearman’s rank-correlations of CP and HI across T2–T5 showed small to moderate positive correlations (Table 2).

**Table 2:** Spearman’s rank correlation between conduct problems and hyperactivity/inattention

	CP T2	CP T3	CP T4	CP T5	HI T2	HI T3	HI T4
CP T2	–						
CP T3	0.45	–					
CP T4	0.33	0.45	–				
CP T5	0.34	0.44	0.54	–			
HI T2	0.39	0.28	0.23	0.23	–		
HI T3	0.27	0.42	0.29	0.27	0.48	–	
HI T4	0.22	0.28	0.42	0.33	0.38	0.53	–
HI T5	0.22	0.31	0.33	0.39	0.37	0.54	0.67

*Note:* CP refers to conduct problems and HI to hyperactivity/inattention as measured by subscales of the Strengths and Difficulties Questionnaire

Caregiver reports and standardised measures collected at nine months were used to examine specific risk markers associated with group membership. Primary caregivers completed questions relating to child sex (boy/girl), their education level (high school or lower/further education), age (16–24/25+), ethnicity (Irish/other), and whether any household member smoked during pregnancy (yes/no). Birth characteristics, also PCG reported, included low birth weight (less than 2500 g, yes/no), stay in the neonatal intensive care unit (yes/no) and premature status (delivery prior to 37 weeks, yes/no). A proxy of family SES was measured using medical card status (cover/no cover), a means tested healthcare benefit provided to low-income households. Social class indicated by profession (managerial and professional level jobs/other or never worked) was also reported by PCGs.

Perceived difficult child temperament was measured using the fussy-difficult subscale of the Infant Characteristics Questionnaire [64] completed by PCGs. This nine-item scale measures caregivers’ perception of whether their baby is fussy and difficult to soothe. Items including *“overall degree of difficulty”* and *“how easily upset”* are assessed using a seven-point scale, with one indicating very easy and seven indicating very difficult. Higher total scores indicate greater perceived difficulty (range: 7–64). This subscale had acceptable internal consistency (0.69) in the GUI sample. The short 8-item Centre of Epidemiological Studies Depression Scale (CESD; [63]) a depression screening tool widely used in research, was also given to PCGs and SCGs. Participants rate how regularly they have experienced depressive symptoms in the last week on a four-point scale (1 = *rarely or none of the time* (less than 1 day), 2 = *some or a little of the time* (1–2 days), 3 = *occasionally or a moderate amount of the time* (3–4 days), 4 = *most or all of the time* (5–7 days). Example items include *“I felt sad”* and *“I felt lonely”*. Total scores can range from 0 to 24, with higher scores indicating higher depressive symptoms. Scores were dichotomised using the cut-off ≥ 7, previously identified as indicating clinically significant depressive symptoms [63]. Internal consistency in the GUI was 0.87.

Caregiver stress was measured using the 18-item Parenting Stress Scale (PSS; [65]). Questions regarding positive and negative experiences of parenting, including *“I am happy in my role as a parent”* and *“The major source of stress in life is my children”*, are rated on a 5-point scale from 1 = *strongly disagree* to 5 = *strongly agree*, with higher scores indicative of greater stress (range: 18–90). Internal consistency in the GUI was 0.74. Parent-child bonding was measured using the Maternal Postnatal Attachment Scale (MPAS) quality of attachment subscale [66]. This nine-item subscale assesses parent’s feelings of pride, affection, enjoyment, patience, and sense of ownership towards the child. This scale was adapted by GUI for SCGs by using five items which are also found on the Paternal Postnatal Attachment Scale (PPAS; [70]). Higher scores indicate greater parent-child bonding. Internal consistency of the MPAS subscale was 0.52 in GUI participants.

### Statistical Analysis

Group-based multi-trajectory modelling (GBMTM) was conducted. This is an extension of group-based trajectory modelling [71], a semi-parametric method for modelling heterogeneity in developmental trajectories. The multi-trajectory extension allows for behaviours to be modelled together, identifying clusters of children following similar patterns of joint CP and HI over time. The identification of distinctive trajectories further allows for the exploration of factors which discriminate between them [72]. The use of a person-centred approach is particularly well suited to examining developmental trajectories of CP and HI, which would not assume a homogenous developmental pattern of linear growth over time.

To identify the best model fit, two, three, four, five, six, seven, and eight-group models were run using a censored norm model. The resulting Bayesian Information Criteria (BIC) and the Akaike Information Criterion (AIC) for each model were compared, whereby larger more positive values represent a better model fit [71, 73–75]. Linear and quadratic growth were then modelled. Whilst the eight-group model had the best fit using the BIC and AIC criteria (see Table 3), further indices of model fit were examined and revealed poor fit. These included the average posterior probability of group membership (APP) and odds of correct classification (OCC). An APP above 70 and OCC above five are considered to indicate a good fit [71]. Both the seven and eight-group models failed to meet the threshold of the APP criteria. Therefore, the six-group model was then examined and provided the best fit for the data using all model fit criteria (see Table 4). A multinomial logistic regression was conducted within the trajectory estimation, keeping the latent structure of the model, to examine risk markers associated with group membership using the no CP/low HI as the reference group. All data was analysed using Stata 17 SE [76], with the statistical threshold set to *p* = < 0.05.

**Table 3:** Model fit: Comparison of the Bayesian informationcriteria and the Akaike information criterion

Model	BIC (n = 54,491)	BIC (n = 7,507)	AIC
2-group	−102992.0	−102977.2	−102925.3
3-group	−101431.4	−101409.6	−101333.5
4-group	−101070.4	−101041.6	−100941.2
5-group	−100698.5	−100662.9	−100538.2
6-group	−100524.0	−100481.4	−100332.6
7-group	−100346.1	−100296.6	−100123.5
8-group	−100246.2	−100189.7	−99992.38

*Note:* The first BIC column represents the total number of assessments used within the estimation of the model across time and participants while the BIC in the second column represents the actual sample size of participants within the estimated trajectories. Together, these two BIC scores bracket the theoretically correct BIC score [71]

**Table 4:** Model fit criteria for joint trajectories of conduct problems and hyperactivity/inattention

Group	N (%)	APP	OCC
1: No CP/low HI	614 (10.6)	82.4	39.6
2: Low-stable CP/HI	835 (14.3)	71	14.6
3: Low-declining CP/stable HI	2042 (35.1)	76.9	6.2
4: Desisting co-occurring CP/HI	1427 (24.5)	78.5	11.2
5: Pure-increasing HI	586 (10.1)	75.6	27.6
6: High chronic co-occurring CP/HI	315 (5.4)	90.2	160

## Results

**Fig. 1:**
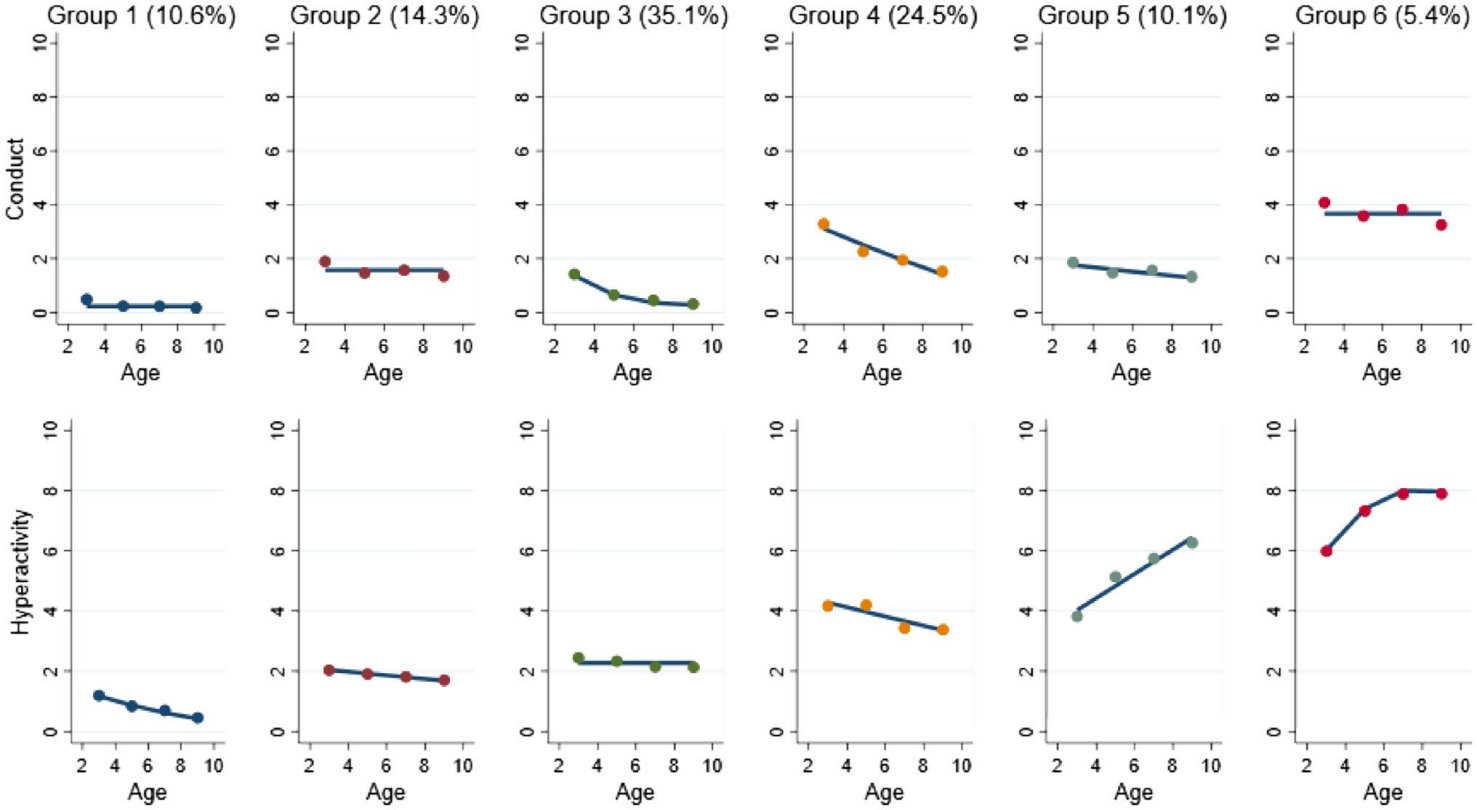
Multi-trajectories of conduct problems and hyperactivity-impulsivity from three to nine years old. *Note:* Group 1 = no CP/low HI. Group 2 = low-stable CP/HI, Group 3 = low-declining CP/stable HI, Group 4 = desisting co-occurring CP/HI, Group 5 = pure-increasing hyperactivity-inattention and Group 6 = high chronic co-occurring CP/HI. Figure shows patterns of CP and HI between three to nine years old in the identified trajectory groups. Group 1 (no CP/low HI), an estimated 10.6% of the sample, characterised by low to no CP and HI which decrease to very low levels at age nine. Group 2 (low-stable CP/HI) an estimated 14.3% of the sample, displayed slightly higher, but still low stable levels of CP and HI. Group 3 (low declining CP/stable HI), an estimated 35.1% of the sample, had low declining levels of CP and low stable HI. Group 4 (desisting co-occurring CP/HI), an estimated 24.5% of the sample, displayed moderate levels of CP which declined to low levels and moderate declining levels of HI between three and nine years. Group 5 (pure-increasing HI), an estimated 10.1% of the sample, was characterised by low declining levels of CP and moderate increasing HI. Group 6 (high chronic co-occurring CP/HI), an estimated 5.4% of the sample, was characterised by high stable CP and high increasing HI

Six groups of children with distinct joint developmental trajectories of CP and HI were identified. Group 1, an estimated 10.6% of the sample, exhibited no CP and low HI at age three, which decreased to very low levels by age nine, and so was labelled as no CP/low HI. Group 2, an estimated 14.3% of the sample, displayed slightly higher, but still low stable levels of CP and HI, and was labelled as low-stable CP/HI. The third group, an estimated 35.1% of the sample, had low declining levels of CP and low stable HI. This group was labelled as low-declining CP/stable HI. Group 4 comprised an estimated 24.5% of the sample and had a moderate level of CP at age three which declined to a low level by age nine, and a moderate declining level of HI between three and nine years. This group was labelled as desisting co-occurring CP/HI. Group 5, labelled pure-increasing HI, comprised an estimated 10.1% of the sample and was characterised by low declining levels of CP and moderate increasing HI. Finally, the sixth group comprised an estimated 5.4% of the sample and had the highest levels of difficulties, characterised by high chronic CP and high increasing HI. Group 6 was labelled as high chronic co-occurring CP/HI. Joint group trajectories can be found in Fig. 1.

To better understand antecedent risk markers associated with membership in elevated trajectory groups (i.e., Groups 4, 5, and 6), a multinomial regression was estimated within the trajectory model using the no CP/low HI as the reference group. Odds ratio (OR) and 95% confidence intervals (CI) for all groups are presented in Table 5. Risk markers associated with membership in the desisting co-occurring CP/HI group (Group 4) at the individual level included child sex (i.e., boys), and being rated by PCGs as having a difficult temperament. At the PCG level, lower age, education level, and higher stress were identified risk markers associated with group membership. At the family level, prenatal exposure to smoking and SES (i.e., non-professional class/managerial jobs) increased the risk associated with group membership. Conversely, higher quality PCG-child bonding and having siblings were protective markers. For Group 5, the pure-increasing HI group, risk markers at the individual level included male sex and being rated by PCGs as having a difficult temperament. Lower PCG education level, higher PCG stress, and prenatal exposure to smoking were also identified as risk markers associated with group membership. Protective markers associated with decreasing the risk of group membership in this group included higher PCG-child bonding and having siblings. Similar risk and protective markers associated with membership in Groups 4 and 5 were associated with membership in Group 6, the high chronic co-occurring CP/HI group, albeit mostly with larger magnitudes of effect size. More specifically, child sex (i.e., boys); difficult temperament (rated by the PCG); lower PCG age, lower education level, higher stress; prenatal exposure to smoking; medical card status; and non-professional class/managerial jobs were all significantly associated risk markers for group membership. Higher PCG-child bonding and having siblings were protective markers.

**Table 5:** Risk factors associated with elevated levels of CP and/or HI: multivariate analysis

	Low-stable CP/HI		Low-declining CP/stable HI		Desisting co-occurring CP/HI		Pure-increasing HI		High chronic co-occurring CP/HI	
	OR	[95% CI]	OR	[95% CI]	OR	[95% CI]	OR	[95% CI]	OR	[95% CI]
Boys	0.93	[0.55, 1.56]	1.83***	[1.33, 2.51]	1.96***	[1.37, 2.80]	5.62***	[3.39, 9.30]	5.17***	[3.34, 8.00]
Born premature	1.25	[0.41, 3.86]	1.22	[0.54, 2.76]	0.77	[0.33, 1.79]	0.86	[0.31, 2.36]	1.02	[0.37, 2.87]
Low birth weight	0.45	[0.11, 1.81]	0.94	[0.33, 2.67]	1.1	[0.39, 3.12]	1.4	[0.44, 4.43]	1.29	[0.37, 4.50]
Neonatal care	0.75	[0.34, 1.67]	1.56	[0.93, 2.63]	1.3	[0.76, 2.22]	1.46	[0.81, 2.65]	1.74	[0.93, 3.26]
Difficult temperament	1.11***	[1.06, 1.16]	1.10***	[1.05, 1.14]	1.16***	[1.11, 1.21]	1.10***	[1.04, 1.16]	1.17***	[1.11, 1.23]
PCG education high school or lower	1.09	[0.59, 2.02]	2.06***	[1.37, 3.10]	2.58***	[1.69, 3.96]	1.98**	[1.23, 3.18]	3.17***	[1.93, 5.22]
PCG age 24 or lower	1.34	[0.23, 7.91]	2.04	[0.59, 7.02]	3.96*	[1.26, 12.52]	2.55	[0.68, 9.52]	4.02*	[1.18, 13.61]
PCG ethnicity non-Irish	1.01	[0.58, 1.75]	0.91	[0.54, 1.55]	1.08	[0.65, 1.80]	0.71	[0.31, 1.62]	0.54	[0.28, 1.06]
PCG depression	0.86	[0.39, 1.87]	0.55	[0.25, 1.20]	1.1	[0.55, 2.20]	1.05	[0.47, 2.34]	1.94	[0.93, 4.02]
PCG quality of attachment	0.87**	[0.79, 0.96]	0.85***	[0.78, 0.93]	0.78***	[0.72, 0.85]	0.83***	[0.75, 0.92]	0.82***	[0.75, 0.91]
PCG stress	1.03	[1.00, 1.07]	1.04*	[1.01, 1.07]	1.09***	[1.05, 1.12]	1.07**	[1.03, 1.11]	1.08***	[1.04, 1.13]
SCG quality of attachment	0.97	[0.86, 1.10]	1	[0.89, 1.13]	0.96	[0.85, 1.08]	0.97	[0.83, 1.12]	0.88	[0.76, 1.01]
SCG depression	1.75	[0.64, 4.80]	1.38	[0.47, 4.05]	1.97	[0.76, 5.10]	1.73	[0.57, 5.26]	1.59	[0.53, 4.80]
SCG stress	1	[0.97, 1.03]	0.99	[0.95, 1.02]	1.02	[0.99, 1.05]	1	[0.96, 1.04]	1.01	[0.98, 1.05]
Exposure to prenatal smoking	1.15	[0.72, 1.84]	1.43	[0.96, 2.14]	1.70**	[1.14, 2.53]	2.08***	[1.36, 3.16]	1.86*	[1.15, 3.00]
Having siblings	1.34	[0.70, 2.56]	0.64*	[0.45, 0.91]	0.59*	[0.39, 0.89]	0.48***	[0.32, 0.72]	0.52**	[0.34, 0.82]
Receiving Medical Card support (lower income)	2.07*	[1.17, 3.69]	1.35	[0.76, 2.40]	1.53	[0.88, 2.67]	1.83	[0.94, 3.57]	2.72**	[1.44, 5.14]
Professional class: non-professional/managerial	1.21	[0.75, 1.95]	1.44*	[1.00, 2.07]	1.70**	[1.19, 2.44]	1.24	[0.77, 1.99]	2.22***	[1.39, 3.56]

*Note:* Group 1 (no CP/low HI) was used as the comparison trajectory group. The reference category for PCG education was tertiary education. The reference category for PCG ethnicity was Irish. The reference category for medical card status was no cover, and finally the reference category for social class was professional/managerial. Odds ratios (OR) and [95% confidence intervals] are presented

* p ≤ 0.05 ** p ≤ 0.01 *** p ≤ 0.001

## Discussion

### Joint CP/HI Trajectory Groups

Grounded in previous findings of single and joint trajectories of CP and HI, we hypothesised that a five-group model would best fit the data. We further predicted that most children would belong to low or moderate-declining (i.e., normative) groups. Additionally, we expected to find groups with moderate to elevated pure CP and pure HI, along with a high chronic co-occurring CP and HI group. Our hypotheses were partially supported. A six-group model best fit the data and included: no CP/low HI, low-stable CP/HI, low-declining CP/stable HI, desisting co-occurring CP/HI, pure-increasing HI, and high chronic co-occurring CP/HI groups. As predicted, most children in this cohort rarely engaged in CP or HI (60%) and a further 24.5% engaged in early moderate but declining levels of CP and HI. Two further groups were identified that had increasing levels of pure HI and chronic-increasing joint CP and HI, comprising a combined 15.5% of the sample. However, the hypothesis of a pure CP group was not supported in this cohort. Given the links between elevated childhood externalising behaviour (i.e., CP and HI) and adverse outcomes across development, the numbers of children identified in this cohort following elevated trajectories of either pure or co-occurring CP and HI is substantial.

These findings largely align with Moffitt’s taxonomy of antisocial behaviour [35]. For example, the high chronic co-occurring CP/HI and desisting co-occurring CP/HI groups appear consistent with the proposed life-course persistent and child-limited groups respectively. However, given that behavioural assessments were only collected up until age nine in the current study, additional measurements of behaviour across adolescence would first be required to confirm the presence of a life-course persistent trajectory group in this cohort. No support was found for a pure CP group in the current study, which may lend further support to the suggestion that children on a life-course persistent trajectory will have coexisting hyperactivity [23]. The literature examining trajectories of CP has yielded mixed results concerning the identification of a pure CP group, whereby some studies have found support [77] and others have not [19, 42, 54, 55]. As co-occurring difficulties were already present at three years of age in this cohort, it is not possible to ascertain whether elevated levels of HI existed prior to elevated CP, as predicted in the taxonomy.

Two groups (i.e., the pure-increasing HI and high chronic co-occurring CP/HI groups) exhibited increasing levels of HI over time, supporting our hypotheses of both a pure and co-occurring group with moderate to high HI starting in early development (i.e., toddlerhood). The increases reported over time may be related to entry into primary school, a key childhood transition which may increase the demands on children to inhibit HI behaviours. Hyperactivity/inattention may increase in children who have not yet developed the skills to inhibit these behaviours during this time as they encounter more situations in which inhibition of these responses is required. This may result in increasing frustration and further subsequent HI. It has also been argued that inattention symptoms will continue to increase in school-age children [53], so the inclusion of an item related to inattention in the SDQ may have also contributed to the observed increases in these groups.

### Antecedent Risk Markers

We found partial support for our hypotheses around associated risk markers for membership in groups with pure and co-occurring elevated CP and HI. At the individual level, for example, and consistent with previous research, boys were more likely to have membership across all elevated groups, with a more than five-fold increase in associated risk for the pure-increasing HI and high chronic co-occurring CP/HI groups. The higher rate of externalising problems in boys has also been linked to other risk factors, particularly higher rates of associated neuropsychological deficits [56]. This is in line with findings in the current study whereby difficult temperament as rated by PCGs was found to be significantly associated with all elevated groups, similar to other studies  [28, 35, 47]. It has been suggested that difficult temperament may also increase the risk of externalising problems through coercive parenting practices which reinforce the child’s behaviour [78, 79]. For example, parents may initially attempt to increase control over their child’s difficult behaviour but give in to their child’s demands as they continue to escalate, reinforcing the behaviour.

Although parent-reported measures of infant temperament are frequently used in research, some debate exists around whether they provide an objective measure of infant temperament or merely measure parental perceptions. Reviews of the literature around the measurement of infant temperament note that studies have found a lack of agreement between parent and observer ratings of infant behaviour and that parent and family characteristics, including personality traits, the presence of mental health difficulties, and lower socioeconomic status have been found to predict higher parental ratings of their child as fussy and difficult (e.g., [80–83]). For the scale used in the present study, modest but significant agreement has been found between maternal and observer ratings of infants, and good agreement between maternal and paternal ratings has also been found [64, 82]. It has been argued that findings such as these suggest that parent-reported measures of infant temperament capture both objective and subjective information [80, 82, 84, 85].

Prenatal exposure to smoking increased the odds of membership across elevated groups, with the largest effect for the pure-increasing HI group (i.e., a greater than two-fold increased risk associated with group membership). Smoking during pregnancy has been associated with CP and HI in multiple studies [2, 34, 35, 58]. This association is suggested to arise from neurobiological changes in brain structure and functioning due to early exposure to nicotine [86]. Additionally, it is argued that smoking during pregnancy may increase the risk of externalising problems in children via complex interactions with other risk factors common to mothers who engage in high-risk activities such as smoking during pregnancy, e.g., having a lower education level and being younger (see [87]). Indeed, many studies have failed to find a robust association between prenatal maternal smoking and externalising problems, suggesting that any association may be an artefact of these additional maternal characteristics (e.g., [88]). Given that our measure of prenatal exposure to smoking included all household members and not just maternal smoking, the results may support a direct link between prenatal exposure to nicotine and increased associated risk of behavioural problems in childhood. Further work examining total exposure in-utero, and not just as a result of maternal consumption, may be warranted.

At the family level, and consistent with past research, lower PCG education level was associated with group membership across all three elevated groups [27, 28, 31, 35, 47]. There is some evidence to suggest that caregivers with lower education levels may have poorer knowledge of parenting practices, poorer communication skills, and lower emotional wellbeing [89, 90], factors which may negatively impact parent-child interactions and increase the risk of CP and HI. Additionally, lower PCG age was associated with membership in the desisting co-occurring CP/HI and high chronic co-occurring CP/HI groups. It may be that younger parents have more limited access to material and personal resources to support them with creating nurturing and supportive environments, which could lead to increased risk for externalising difficulties in children. The lack of association between lower PCG age and the pure-increasing HI group may suggest unique associations for co-occurring externalising difficulties compared to HI difficulties alone.

Indicators of lower SES (i.e., medical card status and social class) were also significant risk markers associated with group membership in groups with co-occurring externalising difficulties (e.g., Groups 4 and 6). The largest associated risk was found for the high chronic co-occurring CP/HI group, wherein being in receipt of the medical card social benefit and having a non-professional/managerial job as the highest level job held by the PCG or SCG were associated with a more than two-fold increase of associated risk of group membership. This finding is consistent with several previous studies that have linked lower SES with elevated developmental trajectories of CP [32, 35] and HI [44]. Moreover, it aligns with Moffit’s taxonomy [23] which suggests that lower SES is a risk factor for life-course persistent groups. It has also been suggested that lower SES, alongside other indicators of social disadvantage such as lower parental education and age, contributes to the development of externalising problems by creating more stressful environments which disrupt family processes and increase the use of coercive parenting practices [91, 92]. Accordingly, some studies have found that the association between SES and child antisocial behaviour is mediated by parenting practices [93, 95].

Primary caregiver stress was also significantly associated with membership in Groups 4, 5, and 6. This is consistent with past research which has found a robust association between higher parental stress and externalising problems in childhood [96–99]. Evidence suggests that interactions between parenting stress and childhood externalising problems may be complex and bidirectional. For example, one longitudinal study of children aged four to ten years found that parenting stress predicted child externalising problems and, in turn, child externalising problems predicted parenting stress over time [100]. Abidin [101] theorised that negative parenting practices mediate the relationship between stress and externalising behaviour in children; however, a number of studies have failed to support this [100, 102–104]. Mechanisms suggested for the direct influence of parenting stress on behavioural problems include that stress creates a negative emotional atmosphere in families [103] and that stressed parents may make use of maladaptive coping strategies such as aggression, which acts to model externalising behaviour to children [104].

### Protective Markers

Previous research has found that children from larger families have an increased risk of CP and HI  [31, 44]. It has been suggested that siblings may contribute to increased externalising problems through coercive interactions, which model and negatively reinforce these behaviours [79]. In particular, sibling relationships characterised by conflict and hostility have been linked with externalising behaviour, especially in sibling pairs who are younger, closer in age, and male [105]. As such, the finding that the presence of siblings was a protective marker associated with membership in the three elevated groups was unanticipated. Some recent studies have found that sibling relationships with high levels of warmth are linked to the reduced likelihood of externalising behaviour [106, 107] and that higher-quality sibling relationships may buffer children against the impact of rejecting parenting [108]. Our findings highlight the need for further research examining the positive effects of sibling relationships and the mechanisms underlying them.

Higher PCG-child bonding, the affective bond or tie the PCG feels towards their child, was also a protective marker for all groups relative to the no CP/low HI group. This replicates similar findings from the Millennium Cohort study, which found that lower self-reported maternal-child bonding was associated with developmental trajectories of elevated externalising behaviour [34]. A lower sense of parent-to-child bonding is theorised to manifest in less responsive and active parenting [66], which is considered fundamental to healthy socioemotional development in children [109]. Accordingly, past research has found that prenatal mother-child bonding is associated with more responsive and sensitive parenting at 12 weeks [110], and that lower maternal responsiveness was associated with increased externalising behaviour in toddlers [111]. In addition, parents experiencing poorer bonding with their children are more likely to experience other risk factors associated with child externalisng problems, including higher stress, and having a child with a difficult temperament [112], which may increase the likelihood of negative parent-child interactions.

### Strengths, Limitations, and Future Directions

To our knowledge, this is one of the few studies to model joint developmental trajectories of HI and CP from toddlerhood to childhood using a person-centred approach, and to investigate early risk and protective markers associated with group membership. The inclusion of under-researched risk markers, including paternal characteristics and parent-child bonding, along with the use of a large nationally representative cohort and well-established measures, are further strengths of the study. Although the present study makes a valuable contribution to the evidence base regarding developmental trajectories of CP and HI, it is not without limitations. First, the use of parent-reports of CP and HI from a single rater (PCGs) is potentially limiting. Future studies would benefit from including multiple raters as this may give a more complete picture of joint developmental trajectories of CP and HI spanning toddlerhood to childhood. Relatedly, information regarding antecedent risk markers were also collected from the same respondent (i.e., the PCG). This increases the likelihood of shared method variance bias being present. Third, data was not yet available to examine the continuation of trajectories into adolescence. Future studies would do well to examine developmental trajectories of CP and HI starting in toddlerhood and extending through to adolescence, an important period of development marked by many transitions and changes. Fourth, attrition across waves, particularly for participants from more disadvantaged backgrounds, could raise issues of generalisability of findings. However, sampling weights were used to bring the distribution of the final sample back to that of the population (i.e., within 0.5%) with respect to participant characteristics. Finally, Cronbach’s alpha for CP was below the desirable threshold (i.e., < 0.70), which may have resulted in an underestimation of trajectory groups and the risk markers associated with group membership [113].

### Clinical Implications and Conclusions

The present findings show that elevated trajectories of co-occurring CP/HI and HI can already be detected in toddlers, and that pre- and postnatal child, parent, and family characteristics can help to differentiate these trajectories from normative trajectory groups. This suggests that at-risk children and their families should be identified and provided with intervention early in life. Such support may be particularly beneficial when targeted to younger mothers with lower educational levels who have overactive sons, and families of lower SES. The contribution of a mix of child, parent, and family risk markers to all elevated groups suggests that interventions should attempt to tackle multiple risk factors. In particular, programmes may benefit from considering the influence of child characteristics and wider social factors, which may restrict parents’ ability to provide optimal care. Given that many of the risk markers associated with elevated trajectories in this study are also associated with lower SES, wider social policy efforts to tackle social deprivation in Ireland could also support efforts to reduce childhood externalising problems. Furthermore, associations between household cigarette consumption during pregnancy and elevated trajectory groups suggest that efforts to reduce smoking during pregnancy should target all household members. These findings also serve to highlight the importance of early parental bonding for later behavioural outcomes. Early years child support services should provide screening and interventions that aim to support early parental bonding with children, particularly for children who experience multiple risk markers associated with chronic co-occurring externalising problems [114].

## Summary

Conduct problems (CP) and hyperactivity/inattention (HI) which develop in toddlerhood are associated with a range of adverse personal and social consequences. CP and HI frequently co-occur, and co-occurring difficulties have been linked both with more severe and persistent problems and related adverse outcomes. This study sought to further understanding of the origins and progression of CP and HI and their potential co-occurrence, by examining joint developmental trajectories of CP and HI in a cohort of 7,507 children in Ireland from ages three to nine, using a person-centred approach (i.e. group-based trajectory modelling), and associated risk/protective markers for trajectory group membership. Data was collected via parent report and standardised assessments. Six trajectories were identified: no CP/low HI, low-stable CP/HI, low-declining CP/stable HI, desisting co-occurring CP/HI, pure-increasing HI, and high chronic co-occurring CP/HI. A combination of pre- and postnatal child, parent, and family characteristics differentiated trajectory groups with elevated CP and/or HI from normative trajectory groups. Results suggest support for both ‘pure’ HI and co-occurring trajectories of CP and HI, which are already observable by toddlerhood. However, a ‘pure’ CP trajectory was not found, which may support the suggestion that children on a persistent CP trajectory will have coexisting HI. Intervention efforts may benefit from starting early in life and targeting multiple risk markers in families with fewer resources. Future research would benefit from examining developmental trajectories of CP and HI starting in toddlerhood and extending through to adolescence, an important period of development marked by many transitions and changes.

## Data Availability

The Growing Up in Ireland Infant Cohort can be accessed from: https://www.ucd.ie/issda/data/guiinfant/.
